# Transgenesis as a Tool for the Efficient Production of Selected Secondary Metabolites from Plant in Vitro Cultures

**DOI:** 10.3390/plants9020132

**Published:** 2020-01-21

**Authors:** Tomasz Kowalczyk, Joanna Wieczfinska, Ewa Skała, Tomasz Śliwiński, Przemysław Sitarek

**Affiliations:** 1 Department of Molecular Biotechnology and Genetics, University of Lodz, Banacha 12/16, 90-237 Lodz, Poland; 2 Department of Immunopathology, Medical University of Lodz, Żeligowskiego 7/9, 90-752 Lodz, Poland; joanna.wieczfinska@umed.lodz.pl; 3 Department of Biology and Pharmaceutical Botany, Medical University of Lodz, Muszynskiego 1, 90-151 Lodz, Poland; ewa.skala@umed.lodz.pl (E.S.); przemyslaw.sitarek@umed.lodz.pl (P.S.); 4 Laboratory of Medical Genetics, University of Lodz, Pomorska 141/143, 90-236 Lodz, Poland; tomasz.sliwinski@biol.uni.lodz.pl

**Keywords:** transgenic plants, secondary metabolites, in vitro plant cultures, metabolic engineering, transgenesis, binary vectors

## Abstract

The plant kingdom abounds in countless species with potential medical uses. Many of them contain valuable secondary metabolites belonging to different classes and demonstrating anticancer, anti-inflammatory, antioxidant, antimicrobial or antidiabetic properties. Many of these metabolites, e.g., paclitaxel, vinblastine, betulinic acid, chlorogenic acid or ferrulic acid, have potential applications in medicine. Additionally, these compounds have many therapeutic and health-promoting properties. The growing demand for these plant secondary metabolites forces the use of new green biotechnology tools to create new, more productive in vitro transgenic plant cultures. These procedures have yielded many promising results, and transgenic cultures have been found to be safe, efficient and cost-effective sources of valuable secondary metabolites for medicine and industry. This review focuses on the use of various in vitro plant culture systems for the production of secondary metabolites.

## 1. Introduction

Throughout history, in his struggle with disease, man has continually enlisted help from plants, one of the richer sources of biologically-active health-promoting substances. The oldest evidence of such use was found on a Sumerian clay slab from Nagpur, which is approximately 5000 years old [1]. However, the use of plants in treating ailments is also mentioned in the Pen T’Sao, written by the Emperor of China, Shen Nung, in 2500 BC, the Indian Vedas, the Ebers Papyrus written in 1550 BC, the Bible and the Talmud [2,3]. Almost all medical systems, be they Traditional Medicine, Kampo medicine, Ayurvedic medicine or European medicine, are based on plant-derived valuable medical compounds [4]. Currently, medicine is faced with a growing demand for a wide range of biologically-active compounds of natural origin that can demonstrate preventive or therapeutic effects against primary causes of death, such as cancer, cardiovascular disease, diabetes or respiratory disease. A large proportion of the phytochemicals used for this purpose are secondary metabolites. These compounds are structurally and functionally-diverse products synthesized in plant cells from various primary metabolites, either constitutively or in response to various stimuli. In nature, they have many different functions such as protecting plants from pathogens [5,6], ultraviolet light [7,8] or herbivores [9,10], and providing specific fragrances and colors to facilitate pollination and seed distribution by animals. They also play an important role as signaling and regulatory molecules for primary metabolic pathways.

In modern medicine, plant secondary metabolites play a vital role. Since their first isolation in 1803 and the introduction of the alkaloid morphine in 1827 [11], studies have expanded knowledge of their structure, biological function, biosynthesis pathway and possible modifications, thanks to which they can be used to protect human health. Many of their mechanisms of action on various types of normal and cancerous human cells have been described [12,13,14]. Secondary metabolites can affect cancer cells by interfering with their division, and by changing their metabolism and even the expression of selected genes. Many also have antioxidant [15,16], anti-inflammatory [17,18], antibacterial [19], antifungal [20,21], neurological [22] or hepatoprotective [23] effects. As plants represent such an important source of many secondary metabolites, there is great interest in increasing their biosynthetic rates as part of green biotechnology, which includes the use of transgenic plants or other photosynthetic organisms for industrial purposes. Such approaches allow the production of a wide range of products including secondary metabolites, recombinant proteins, biologically-active compounds, paper and biofuels. They can also be used to improve the nutritional quality of a plant and the development of environmentally-friendly farming solutions.

One of the key strategies enabling the overproduction of valuable plant secondary metabolites in in vitro cultures is the manipulation of existing metabolic pathways by overexpressing or silencing selected elements involved in their biosynthesis [24,25,26,27]. A wide range of plant vectors have been designed that allow the simple and quick introduction of synthetic expression cassettes, allowing easier and more effective creation of in vitro transgenic plant cultures [28,29,30]. Several studies confirm that such modulation of metabolic pathways increases the yield of naturally-occurring biologically-active compounds with potential use in medicine [31,32,33].

The aim of this work is to review the latest achievements in the transgenesis-based biosynthesis of selected secondary metabolites, particularly anticancer compounds, produced in various types of in vitro plant cultures.

## 2. In Vitro Plant Cells and Organ Cultures as an Alternative Source of Secondary Metabolites

Since the development of the concept of in vitro cell culture in 1902 by the German physiologist Gottlieb Haberlandt, it has been possible to cultivate cells, organs or whole plants of many species under strictly controlled conditions; such approaches employ various biotechnological methods to exploit the totipotency (natural ability to divide and produce differentiated cells) of plant tissue. At present, in vitro plant cultures have been used for agricultural purposes by clonal multiplication of plants [34] and the introduction of novel genetic variations and transgenic plants. They have also found use in pharmaceutical biotechnology, through the production of recombinant proteins, monoclonal antibodies and secondary metabolites, and environmental biotechnology, by developing new methods of eliminating ecological threats and phytoremediation. Plant cell and tissue culture offers many advantages, including the ability to maintain the cells under controlled conditions regardless of changes in environmental conditions, simple extraction of biologically-active compounds, efficient production of many valuable compounds, the ability to easily scale the production process, and faster reproduction of high-quality plant material, especially in the case of slow-growing plants or those producing a small number of seeds. Additionally, this strategy allows pathogen-free material with high levels of the desired biologically-active compounds to be produced. Typically, calli, cell suspension cultures, roots or shoots are most commonly used for obtaining secondary metabolites in vitro. In selected cases, it is necessary to induce cell differentiation in cultures because some metabolites can be synthesized only in specialized plant tissues or glands (e.g., essential oils).

Each of the aforementioned types of in vitro plant cultures has certain characteristic features making it suitable for a chosen group of compounds. All types of cultures have their advantages and disadvantages, and care should be taken to select an appropriate type of plant culture to obtain valuable secondary metabolites in the first stage. 

## 3. From Natural Gene Transfer to Plant Metabolic Engineering

Over 100 years have passed since the first isolation of *Agrobacterium tumefaciens*, which later turned out to be a natural genetic engineer of the plant genome [35]. *A. tumefaciens* is a phytopathogen found in an environment causing neoplastic diseases (crown gall) on various plant species. The bacterium has a natural ability to infect wound sites in plants, resulting in the formation of characteristic tumors [36] by the transfer of T-DNA from the bacterial cell to the plant genome through a bacterial type IV secretion system (T4SS) [37]. The first use of *A. tumefaciens* infection to obtain transgenic plants was in 1983 [38], which predicted the advent of a new age in plant biotechnology. As the mechanism of transferring genetic material to the plant cell became better understood, such transfection by *Agrobacterium* has become the most popular method of genetic modification of plants. During transformation, T-DNA is exported from the bacterial cells to the nucleus of the plant cell, where it is integrated into the chromosomal DNA. Interestingly, when infecting plant tissue, *Agrobacterium* has the ability to inhibit the plant’s natural defense response [39].

The molecular mechanism of genetic transformation using *Agrobacterium* is well understood. It is known that the transfer of genes from a bacterial cell to a plant cell is based on the transfer of T-DNA, which is part of the Ti megaplasmid. This motile genetic element is responsible for tumor induction and biosynthesis of opines in plant tissue. It contains two regions associated with bacterial–plant interactions: *vir* regions, containing virulence genes (*vir*A*, vir*B, *vir*C, *vir*D, *vir*E, *vir*G, *vir*F and *vir*H) encoding proteins actively involved in the transfer and integration of the transgene with the plant genome, and a region containing genes coding the synthesis of inter alia opines, which are used as a source of carbon and nitrogen by bacteria [40]. This procedure allows the creation of transgenic plants by so called stable transformation, in which the new trait is inherited by further generations, or transient transformation, in which the genetic material persists in the cell nucleus without permanent integration with the genetic material. Since the first successful attempts at genetic transformation of plants by *Agrobacterium* in the early 1980s, the system has demonstrated great potential in transforming dicotyledonous and monocotyledonous plants.

However, other ways exist for changing the plant genome. Such chemical methods include treatment of protoplasts with polyethylene glycol (PEG), facilitating stable and transient transformation [41]. Transformation can also be made more effective by electroporation: the creation of transient micropores in the cell membrane by an electrical impulse to allow the passage of DNA into protoplasts [42]. Finally, microprojectile bombardment can be used, in which particles of gold or tungsten are coated with the appropriately prepared DNA molecules and pushed in the cell by high voltage or compressed gas [43].

Plant genetic transformation has a very wide application in expression of recombinant proteins [44]; these can have many therapeutic or diagnostic uses, increase plant resistance to biotic and abiotic stresses [45] or increase their nutritional or taste values [46]. Another approach is known as metabolic engineering: it can be used to increase the production of selected metabolites that are naturally synthesized in their tissues, or for the synthesis of completely new compounds [47,48].

## 4. Binary Vectors as a Basic Tool in Plant Genetic Transformation

**Figure 1:**
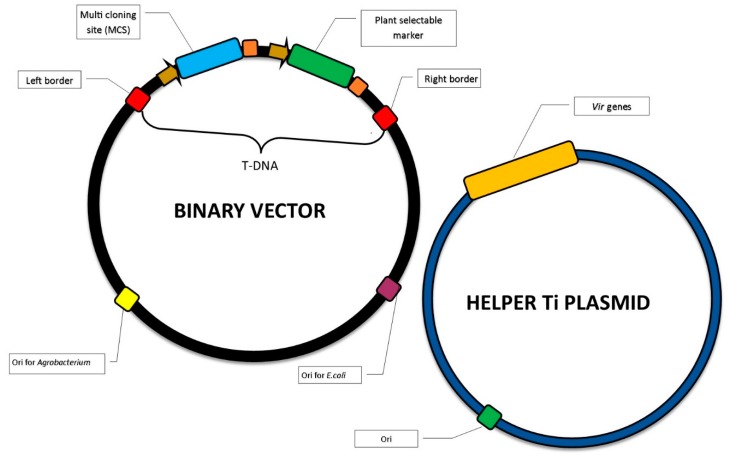
Schematic representation of binary and helper vectors used in plant genetic transformation.

New developments in genetic engineering have allowed much greater control over the transfer of foreign genes to plant cells. A number of vectors can now be used in transgenesis [28,49] that enable different marker and reporter genes and restriction sites to be chosen; they also allow high copy numbers of the binary vectors already in common use in biotechnological laboratories in bacterial cells. *Agrobacterium*- based transformation typically proceeds according to the following process: first, naturally-occurring oncogenes are removed to deprive the bacterium of pathogenicity; such a strain is often referred to as *disarmed*. The genes of interest are added to the T-DNA, together with appropriate selection or reporter genes. Due to its large size, the Ti (tumor inducing) or Ri (root inducing) plasmid does not replicate in *Escherichia coli* and hence only occurs in bacterial cells in low copy numbers, thus complicating its isolation; to allow easier manipulation, many works are carried out with a so-called binary vector system. The idea of this approach is to separate *vir* and T-DNA regions into two independent replicons. The T-DNA is located on a binary vector containing the origin of replication for *E.coli* and *Agrobacterium*. This vector also contains left and right borders designating the T-DNA region, selection genes for bacterial and plant cells, and a number of specific sites recognized by restriction enzymes allowing for easy cloning (Figure 1).

## 5. Calli and Cell Suspension Cultures 

Under natural conditions, callus tissue is formed in the plant at the site of its injury from surrounding cells to seal wound sites and prevent water loss [41]. In laboratory conditions, this cell mass is induced on artificial media by using plant growth regulators (mainly auxins and cytokinins). This type of plant culture can be successfully used for the regeneration of whole plants (embryogenic callus) or as a material for establishing plant suspension cultures. One strategy for obtaining selected secondary metabolites involves the use of medicinal plant cell suspension culture (MPCSC), where single cells or different sized aggregates of cells are cultured in agitated liquid medium.

Calli cultures are often used successfully to produce secondary metabolites of medical significance, many of which may be used in treating human diseases [50,51,52,53]. The synthesis of the desired compound can be increased by designing the genetic constructs and the way they are introduced. An example would be to induce overexpression of three stilbene synthase (STS) genes of *Picea jezoensis* (Siebold & Zucc.) Carr, *viz. PjSTS1a*, *PjSTS2* and *PjSTS3*, in calli cultures of *Vitis amurensis* Rupr., resulting in an increase in the content of stilbene [54]. Another example is the overexpression of 1-deoxy-d-xylulose-5-phosphate synthase 1 (*SrDXS1*) and kaurenoic acid hydroxylase (*SrKAH*) in *Stevia* spp., which increases the production of steviol glycosides [55], or the overexpression of stilbene synthase (*VaSTS7*) to increase production of resveratrol in transgenic cell cultures of *V. amurensis*. 

Another good source of secondary metabolites is by the use of transgenic cell suspension cultures. One example is the stable transformation of *Silybum marianum* (L.) Gaertn. cell suspension cultures with the *Vitis vinifera* L. stilbene synthase gene, allowing increased accumulation of t-resveratrol [56]. In addition, overexpression of the neutral / alkaline invertase (*NINV*) gene in *Taxus chinensis* (Rehder & E.H.Wilson) Rehder cell suspension significantly enhances the expression of the taxadiene synthase (*TAS*) gene, and the biosynthesis of seven individual taxanes [57].

## 6. Hairy Roots 

Hairy root cultures arise as a result of infection of plant tissue by *Agrobacterium rhizogenes*, resulting in the unlimited growth of organized plant tissue. The ability of these bacteria to infect the plants is due to having large plasmids known as Ri plasmids (root inducing) that contain transfer *rol* (*rol*A, *rol*B, and *rol*C) genes responsible for the stable integration of genetic material into host cells DNA. For many reasons, this system of obtaining valuable biologically active compounds is very convenient. Hairy roots are fast growing, require no plant growth regulators, are highly genetically stable and are able to grow on a large scale [58]. Considering all the advantages of hairy roots over other types of in vitro plant culture systems, they seem to be the best transgenic alternative to medicinal plants occurring in the natural environment. Hairy roots produce relatively large amounts of biologically-active compounds without interference from the natural environment. In addition, the ability to change their metabolic pathways through genetic engineering puts them at the forefront of currently used in vitro plant cultures.

Hairy root cultures have an extremely wide range of applications in green biotechnology [59,60,61]. As well as production of recombinant proteins, biotransformation or phytoremediation, they are a rich source of many valuable secondary metabolites, especially those produced by medical plants. As a plant production system, hairy roots offer many advantages over bacterial or mammalian systems: they have the ability to synthesize many compounds that are difficult to synthesize chemically, while the ability to grow and develop on simple media makes them attractive for economic reasons. There is no risk of transmission of human or animal pathogens, which is very important for medicinal compounds. So far, many attempts have been made to induce hairy roots with specific traits, most of which are based on genetic engineering techniques that allow plant material with increased utility values to be obtained. A good example of this approach is the heterologous expression of *Vitreoscilla* hemoglobin in plastids using the pVHb-RecA construct, leading to increased production of hyoscyamine and scopolamine in *Hyoscyamus niger* L. in vitro transgenic plant cultures [62]. Another is the possibility of affecting the increase in the content of glycyrrhizic acid by overexpression of the β-amyrin synthase gene in hairy roots of *Glycyrrhiza uralensis* Fisch. ex DC. In this work, a genetic construct containing the tobacco root-specific promoter *TobRB7* and *GuBAS* cDNA was used [63].

## 7. Selected Secondary Metabolites in Medical Use Obtained by in Vitro Transgenic Plant Culture

### 7.1. Anticancer Compounds

Cancer is a serious disease that causes the deaths of many people around the world every year [64] and new therapies are constantly being sought. In this context, compounds of natural origin, including many plant-derived chemicals, including paclitaxel, vinblastine, vincristine and camptothecin, play an extremely important role in prophylaxis and therapy [65,66,67]. These substances are often safer and less toxic than synthetic ones [68]. Great effort has been invested in increasing their production, and this has resulted in the design of a range of biotechnological methods to increase productivity.

### 7.2. Paclitaxel

Paclitaxel is an anti-cancer drug originally isolated from the bark of the Pacific yew tree, *Taxus brevifolia* Nutt. in 1971 [69]. It is known to act on microtubules, i.e., the structures responsible for the formation of the mitotic spindle during cell division and cytoplasmic movement in the cell; more specifically, the drug promotes the assembly of microtubules from tubulin dimers and stabilizes microtubules by preventing depolymerization. It thus blocks metaphase–anaphase transitions, inhibits mitosis, and induces apoptosis in a wide range of cancer cells [70].

The drug is used in the treatment of a range of conditions including lung, ovarian, breast, stomach, esophageal, cervical, and prostate cancer, as well as lymphoma and leukemia [70]. As this drug is widely used in cancer therapy in humans, there is great interest in increasing its productivity from natural sources. Although paclitaxel can be chemically synthesized [71], this process is not commercially profitable, and its best sources are in vitro and ex vitro plant cultures. A number of previous works have attempted to optimize the process of obtaining taxanes by genetic transformation. Some strategies have resulted in increased paclitaxel production: overexpression of 10-deacetylbaccatin III-10-O-acetyltransferase (DBAT) and taxadiene synthase (TXS) in transgenic *Taxus marei* (Lemee & H.Lev.) [72] or TXS in *Taxus* x *media* Rehder var. *hicksii* cell culture [73]. Other studies have found that enhancement of paclitaxel biosynthesis can be obtained by overexpression of the 9-cis-epoxycarotenoid dioxygenase gene in transgenic cell lines of *T. chinensis* [70]. In addition, genetic transformation of *Nicotiana benthamiana* Domin with a taxadiene synthase (TS) gene under control of the 35S Cauliflower Mosaic Virus (CaMV 35S) promoter was found to enable de novo production of taxadiene in *N. benthamiana* homozygous lines, yielding 11–27 μg taxadiene/g of dry weight; in addition, subsequent elicitor treatment (methyl jasmonate) increased taxadiene accumulation by a further 1.4 times [74]. A similar approach based on the in vitro transformation of *T*. x *media* hairy roots and subsequent elicitation allowed the production of paclitaxel; the vector was *A. tumefaciens* carrying the RiA4 plasmid and the binary vector pCAMBIA-TXS-His harboring the taxadiene synthase (*txs*) gene of *Taxus baccata* L. under the control of the 35S CaMV promoter [75].

### 7.3. Camptothecin

Camptothecin (CPT) is a monoterpene alkaloid and potent inhibitor of topoisomerase I (Top 1): a nuclear enzyme involved in DNA repair, recombination, transcription and replication [76]. This compound and several of its derivatives, such as irinothecan or topothecan, are in clinical use against a number of human cancers. In vitro plant cultures have long been used as a source of camptothecin, with the first works, in which Sakato et al. [77] presented the possibility of acquiring this compound from *Camptotheca acuminate* Decne cell suspension cultures, dating from the 1970s. 

Since these initial studies, more focused attempts have been made to increase camptothecin production using modern biotechnology in plant cells, for example, overexpression of the *ORCA3* gene in transgenic hairy root lines [78] and allene oxide cyclase from *C. acuminate* [79].

Cui et al. [80] obtained *Ophiorrhiza pumila* Champ. ex Benth hairy roots with separate or simultaneous overexpression of the transformed *Catharanthus roseus* (L.) G. Don genes for strictosidine synthase (*STR*) and geraniol 10-hydroxylase (*G10H*). Their findings clearly show greater accumulation of CPT in transformed *O. pumila* hairy roots. Co-overexpression of the *G10H* and *STR* genes resulted in a 56% increase in camptothecin accumulation compared to non-transgenic HR lines. In addition, a comparison of 25 hairy root lines of *Ophiorrhiza mungos* L., obtained via *A. rhizogenes* transformation, confirmed elevated camptothecin production in the hairy roots (0.32% CPT of DW) compared to non-transformed cultures (0.25% CPT of DW) [81]. Wang et al. also reported significantly greater total production of camptothecin in selected transgenic hairy roots of *O. pumila*. Transformation was performed using a genetic construct encoding tryptophan-arginine-lysine-tyrosine (WRKY), *OpWRKY3* isolated from *O. pumila*, which has high homology with the *VvWRKY30* factor [82]. These results further demonstrate the potential of transgenesis to modulate the productivity of plant cells.

### 7.4. Vincristine

Vincristine is one of the first plant alkaloids approved by the FDA for use in the treatment of cancer. In nature, it occurs in the leaves of *Catharanthus roseus* (L.) G.Don and has been used for a long time in various branches of medicine, including pediatric oncology, as an effective drug against lymphoblastic leukemia [83], rhabdomyosarcoma [84], neuroblastoma [85], Hodgkin lymphoma [86] and Wilms tumor lymphomas [87]. The mechanism of its anti-tumor activity is based on its prevention of microtubule formation by binding to tubulin. As a consequence, mitosis is halted in metaphase following disruption of mitotic spindle formation. The compound also has the ability to inhibit the synthesis of nucleic acids and proteins [88]. 

Canel et al. [89] overexpressed two genes coding for tryptophan decarboxylase and strictosidine synthase in callus and leaf tissue of *C. roseus* via *A. tumefaciens* LBA 1119 transformation. Pham et al. [1] also conducted an agrotransformation of periwinkle using a *CrDAT* transgene encoding deacetylvindoline 4-O-acetyltransferase (DAT): a key enzyme that catalyses the formation of vinblastine and vincristine. The authors demonstrated that selected *C. roseus* lines are capable of higher accumulation of vincristine through overexpression of the DAT protein, involved in the biosynthesis of terpenoid indole alkaloids (TIAs); in this case, the DAT gene itself was under the control of the CaMV 35S promoter. Transformation resulted in 1.63- to 2.48-fold greater production of vincristine compared to non-transgenic plants. These results show that the expression of DAT can significantly affect the accumulation of vincristine in *C. roseus.*

### 7.5. Vinblastine

Vinblastine has a similar mechanism of action to vincristine and is also a widely-used plant alkaloid in cancer therapy. Similar to other valuable plant metabolites, vinblastine is also of interest to modern plant biotechnology and many studies have attempted to increase its production in plant tissues. One study examined the effect of overexpression of the MYC1 transcription factor (CrMYC1) in *C. roseus* [90]. CrMYC1 has been characterized as one of the main components regulating the biosynthesis of terpenoid indole alkaloid metabolites in this plant. Briefly, the authors cloned the CrMYC1 coding sequence into a plant binary vector and then transiently expressed the gene in *C. roseus* by agroinfiltration. The resulting overexpression of this transcription factor increases the level of important terpenoid indole alkaloids such as vinblastine, vincristine or catharantine. The results showed a 2.5-fold increase in vinblastine production and a 3-fold increase in catharanthine relative to control plants. Another study employed transient overexpression of CrERF5 (AP2/ERF transcription factor) in *C. roseus* petals to increase the expression of key genes in the monoterpene indole alkaloid (MIA) biosynthesis pathways [91]. The transformation led to an increase in the content of the bisindole alkaloids anhydrovinblastine and vinblastine, and the monoindole alkaloids ajmalicine, vindoline and catharanthine.

## 8. Overproduction of Other Secondary Metabolites in Transgenic in Vitro Cell Culture 

Each class of secondary metabolites is formed by a complex network of precursors, enzymes and co-factors, some of them leading to specific plant-derived medicinal compounds. In addition to the main anti-cancer alkaloids, which have been presented above, a number of other terpenes, or phenolic compounds, which are common in many other plant species of the *Lamiaceae, Asteracea* and *Fabaceae* families, among others, are of potential value. Many such compounds are known to possess various anti-inflammatory, anticancer, antioxidant, antidiabetic, hepatoprotective or antimicrobial properties [15,16,17,18,19,20,21,22,23]. Of these, the antibacterial properties have drawn significant interest due to the growing problem of bacterial infections around the world [92,93]. These compounds are known to disrupt membrane function and structures, interrupt DNA or RNA synthesis and function, and interfere with intermediary metabolism or intercellular communication via various mechanisms of action [92,94,95]. Importantly, the prevalence of multi antibiotic-resistant bacterial strains in the environment is growing [96,97]. A good example of the potential of transformation to improve the biosynthetic efficiency of antibacterial metabolites can be seen in transgenic *Codonopsis lanceolata* (Siebold & Zucc.) Beneth. and Hook.f. ex Trautv. over-expressing the γ-tocopherol methyl transferase (*γ-tmt*) gene, which leads to increased antimicrobial activity against gram-positive and gram-negative bacteria compared to controls. The plant was transformed with a genetic construct containing γ-tmt cDNAs from *Arabidopsis thaliana* (L.) Heynh. under the control of CaMV 35S promoter and NOS terminator [98]. In turn, Ghimire et al. [99] report that overexpression of the *γ-tmt* gene in *Perilla frutescens* L. increases the levels of phenolic compounds (gallic acid, pyrogallol, 5-sulfosalicylic acid, catechin, chlorogenic acid, vanillin, syringic acid, naringenin, salicylic acid, quercetin, *o*-coumaric, kaempferol, hesperetin and benzoic) in the transgenic plants, and that this elevated phenolic content was associated with stronger antimicrobial activity in comparison to wild plants.

Other broad-spectrum metabolites can be modified to increase the production of specific molecules in in vitro plant cultures. For example, over-expression of the AtPAP1 transcriptional factor was found to enhance phenolic acid (such as chlorogenic acid, caffeic acid, ferulic acid or *p*-coumaric acid) production in a transgenic root culture of *Leonurus sibiricus* L., and the transgenic roots with the AtPAP1 transcriptional factor demonstrated better antimicrobial potential and cytotoxic activity against grade IV glioma cells [100]. Other studies have found such transgenic roots, incorporating the AtPAP1 transcriptional factor, to demonstrate better anticancer effects vis DNA damage, PARP cleavage/increased H2A.X histone levels and UHRF-1/DNMT1 downregulation of mRNA levels compared to untransformed roots [101]. In addition, the extract derived from transgenic *L. sibiricus* roots overexpressing AtPAP1 demonstrated a stronger cytotoxic effect against melanoma cells and had a higher antioxidant potential in human blood plasma [102]. In turn, better cytotoxic and genotoxic effects were demonstrated against acute lymphoblastic leukemia (CCRF-CEM) and chronic myelogenous leukemia (K562) cell lines after treatment with extract from AtPAP1 transformed roots, possibly due to its higher phenolic acid content [103]. 

A representative terpene is betulinic acid, a pentacyclic triterpenoid, which is gaining considerable attention due to its unique anti-cancer activity, allowing selective inhibition of melanoma growth without damaging normal cells [104]. Its presence was initially reported in *Betula* spp., *Ocimum* spp., *Senna* spp. and *Menynathes* spp., among others. Suzuki et al. revealed that metabolic modification of *Lotus japonicus* L. by changes in *CYP716A51* expression may increase betulinic acid biosynthesis [105]. These studies confirm that biotechnology techniques are legitimate tools for increasing the production of secondary metabolites in in vitro plant cultures. Other examples of metabolic pathway manipulation to increase secondary metabolite content in in vitro plant cultures are presented in Table 1 below.

## 9. In Vitro Transgenic Plant Cultures and Societal Implication

The intensive pace of recent scientific progress has been accompanied by a similar growth in public interest regarding issues related to health protection and the treatment of civilization diseases. In addition, with the growth in awareness of the dangers associated with a polluted environment, and the measurable economic benefits associated with productive agriculture, comes a greater willingness to accept research on genetic modification. With this in mind, the metabolic engineering of plant cells is an effective way to obtain valuable biologically-active compounds for pharmaceuticals. Today, thanks to modern biotechnology, it is possible to produce many therapeutics, even on an industrial scale.

Plant-based genetic manipulation is regarded as far more acceptable than such work with animals. Humans have modified plant genomes using conventional methods (crossing) for thousands of years. In addition, as the use of in vitro transgenic plant cultures is usually limited only to the laboratory, they do not have such negative connotations as the production of transgenic crops. In addition, given the appropriate cooperation of the scientific community, such research enjoys quite high social acceptance. Most importantly, green biotechnology can significantly contribute to achieving many of the sustainable development goals (SDGs) set in 2015 by the United Nations, these being intended to cover a broad range of global social and economic development targets by 2030 [149].

## 10. Conclusions

Many compounds currently used in medicine are of plant origin, and the literature suggests a growing tendency to return to biologically-active compounds of natural origin. In contrast to chemically synthesized compounds, many compounds from a natural origin show greater biological safety, generate fewer side effects and are often characterized by lower production costs. However, due to their limited adaptability, the diversity of medicinal plant species in their natural environment has been shrinking the face of intense and unfavorable climate change and growing anthropogenic environmental pollution. In response to this, and the constantly growing demand for compounds of plant origin, new and more efficient in vitro techniques for growing plants are being developed. The literature reviewed showed that in combination with the currently-available precision tools of molecular biology and genetic engineering, high-throughput in vitro plant cultures can be in some cases used to provide many natural secondary metabolites. Modern green biotechnology, which allows manipulation of cellular processes at many levels, can be an excellent alternative to traditional methods of obtaining biologically-active compounds. The ability to create various genetic constructs and introduce them into the plant genome can be an efficient production platform for a wide range of compounds used in medicine, diagnostics or industry. Currently, intensive work is underway on new biotechnological solutions and sustainable alternative methods of producing high-value plant metabolites.

## Figures and Tables

**Table 1:** Secondary metabolites derived from genetically-modified plant in vitro cultures with their biological properties.

Plant Species	Vector/Genetic Construct	Plant Material	Extraction Solvent	Class of Compounds	Effect	References
*Atropa belladonna* L.	pXI vector containing NtPMT and HnH6H	Whole plant	methanol and acetate acetate (methanol:50mM ammonium acetate = 58:42)	Alkaloids	Enhanced biosynthesis of scopolamine	[106]
*Papaver somniferum* L.	pTRV2-BBE, pTRV2-COM, pTRV2-BBECOM	Leaves	Methanol	Alkaloids	Changes in the different alkaloids content	[107]
*Arabidopsis thaliana* (L.) Heynh.	pBI121 vector containing UGT76E11	Seedlings	Methanol	Polyphenols	increased content of flavonoid glycosides (kaempferol 3-O-[6″-O-(rhamnosyl) glucoside] 7-O-rhamnoside kaempferol 3-O-glucoside 7- O-rhamnoside, kaempferol 3-O-rhamnoside 7-O-rhamnoside quercetin 3-O-rhamnoside 7-O-rhamnoside and quercetin 3-O-glucoside 7-O-rhamnoside)	[108]
*Arabidopsis thaliana* (L.) Heynh.	35Spro: AtUGT78D1	Seedlings	Methanol	Polyphenols	Increased accumulation of flavonoids	[109]
*Arabidopsis thaliana* (L.) Heynh.	pCAMBIA1301-AtMYB12	Seedlings	HCl-methanol	Polyphenols	Increased content of flavonoids	[110]
*Arabidopsis thaliana* (L.) Heynh.	pCAMBIA1301- AeCHS	Seedlings	HCl-methanol	Polyphenols	Increased level of flavonoids	[111]
*Arabidopsis thaliana* (L.) Heynh.	pCAMBIA1301-AmDEL	Seedlings	HCl-methanol	Polyphenols	Increased level of flavonoids	[112]
*Ipomoea batatas* (L. Poir.)	pCam-SPO-IbMYB1a	Storage root	Methanol	Polyphenols	Increased anthocyanin content	[113]
*Ipomoea batatas* (L. Poir.)	pGWB11 vector containing IbOr	Storage root	HCl-methanol	Polyphenols	Enhanced accumulation of zeaxanthin and β-carotene	[114]
*Leonurus sibiricus L.*	pCAMBIA1305.1-AtPAP1	Hairy roots	Methanol-water	Polyphenols	Higher phenolic acid content. In addition, tested extracts with higher amounts of phenolic acids showed better antimicrobial and cytotoxic effect.	[102]
*Linum usitatissimum* L.	pBinAR	Whole plants	HCl-methanol	Polyphenols	*Increased level of flavonoids*	[115]
*Nicotiana benthamiana* Domin	pMV-EsMYBF1	Flowers	HCl-methanol	Polyphenols	Increased production of flavonol content	[116]
*Nicotiana tabacum* L.	pGR-STS and pGR-ROST	Whole plant	80% metanol	Polyphenols	Increased production of resveratrol derivatives (piceid, piceid methyl ether, resveratrol methyl ether O-hexoside, and 5-methyl resveratrol-3,4-O-β-d-diglucopyranoside)	[117]
*Nicotiana tabacum* L.	pK2GW7 vector containing NtFLS2	Leaves	Methanol-water-chloroform (5:2:2)	Polyphenols	Increased accumulation of rutin	[118]
*Nicotiana tabacum* L.	pCambia1305 containing SbMYB8	Leaves	Ethyl alcohol	Polyphenols	higher caffeoylquinic acid contents	[119]
*Nicotiana tabacum* L.	pZIP-Bar containing PgDDS, CYP716A47 and UGT71A28	Leaves	100% methanol	Polyphenols	Enhanced production of ginsenoside saponin	[120]
*Nicotiana tabacum* L.	pSAK277 vector containing 35S:StMYBA1-1 construct	Leaves	HCl-methanol	Polyphenols	Enhanced anthocyanin accumulation	[121]
*Petunia* x *hybrida hort. ex E.Vilm*	pBI-121 containing Fh3GT1	Blooming flowers	HCl-methanol	Polyphenols	Increased production of cyaniding, peonidin derivatives, kaempferol derivatives and quercetin derivatives	[122]
*Petunia* x *hybrida hort. ex E.Vilm*	pB7WG2D vector containing RsMYB1	Leaves	HCl-methanol	Polyphenols	Enhanced accumulation of flavonoids	[123]
*Salvia miltiorrhiza* Bunge	pCAMBIA2300 vector containing SmANS	Plantlets	HCl-methanol	Polyphenols	Increased anthocyanin accumulation, flavonols and proanthocyanidins biosynthesis	[124]
*Salvia miltiorrhiza* Bunge	pCB2006-EDT1	Roots	80% methanol	Polyphenols	Increased accumulation of salvianolic acids	[125]
*Salvia miltiorrhiza* Bunge	pEarleyGate201–SmMYC2	Roots	75% methanol	Polyphenols	Enhanced production of hydrophilic phenolic acids	[126]
*Salvia miltiorrhiza* Bunge	pEarleyGate202-SmJMT	Roots	Methanol-acetone (7:3)	Polyphenols	Increased production of salvianolic and rosmarinic acids	[127]
*Solanum lycopersicum* L.	pE1775-CHI pDEL.ROS	Flesh and peel	HCl-methanol	Polyphenols	Enhanced anthocyanins and flavonols	[128]
*Solanum lycopersicum* L.	K303 vector containing SlMYB75	Fruits	80% methanol	Polyphenols	Increased accumulation of anthocyanin, phenolics and flavonoids	[129]
*Solanum melongena* L.	pBIN19+SmHQT pBIN19+p19	Fruits	Methanol-water (80:20)	Polyphenols	Increased level of phenolic compounds	[130]
*Taraxacum brevicorniculatum* Korol.	pBI-AtPAP1	Leaves	Methanol and formic acid	Polyphenols	Increased production of anthocyanins, phenolic acids and flavonoids	[131]
*Trachyspermum ammi* L. Sprague	pBI121-TP	Leaves	80% ethanol	Polyphenols	Increased production of thymol	[132]
*Withania somnifera* L.	pYL436 vector containing Ws-Sgtl4	Hairy roots	Methanol	Steroids	Increased withanolide and withanolide-A contents	[133]
*Artemisia annua* L.	pCAMBIA1305–DBR2	Leaves	Methanol	Terpenoids	Increased level of artemisinin	[134]
*Artemisia annua L.*	pIG-TfGA20ox2	Leaves	Methanol	Terpenoids	Increased production of artemisinin, sesquiterpenes. Eucalyptol, borneol, α-caryophyllene, β-guaiene, δ-cadinene and β-cubebene and isomultiflorenone were detected only in transgenic extract	[135]
*Betula platyphylla*	pSGRNAi-GSNOR	Cell suspension or plantlet stems	Ethanol	Triterpenoids	Increased betulin content	[136]
*Citrus grandis* L.	pK2- CsMADS6	Calli and fruit	-	Terpenoids	increased carotenoid contents	[137]
*Lavandula latifolia* Medik.	pBILIS	Leaves	Hexane	Triterpenoids	Increased production of terpenes (S-linalool)	[138]
*Mentha spicata* L.	pK7WG2D- MsYABBY5	Leaves	Ethyl acetate	Triterpenoids	Increased production of terpenes by gene silencing	[139]
*Mentha spicata L.*	pB1121 vector containing IPP	Whole plants	-	Terpenoids	Increased production of terpenoids	[140]
*Nicotiana tabacum* L.	pSKAN35SGES	Leaves	Methanol	Triterpenoids	Increased production of terpenes	[141]
*Panax ginseng* CA Meyer	pCAMBIA1390 vector containing PgLOX6	Roots	80% methanol	Terpenoids	Increased production of ginsenosides	[142]
*Pelargonium graveolens* L’Her *and Withania somnifera* (L.) Dunal	pBI121 vector containing GrDXS	Whole plants	-	Terpenoids	Increased production of essential oil and withanolides	[143]
*Salvia miltiorrhiza* Bunge	pBI121 vector containing SmMDS	Hairy roots	80% methanol	Terpenoids	increased accumulation of tanshinones (dihydrotanshinone I, cryptotanshinone, tanshinone I andtanshinone IIA)	[144]
*Salvia miltiorrhiza* Bunge	pCAMBIA2300sm-SmWRKY2	Hairy roots	Methanol/di- chloromethane (3:1)	Terpenoids	Increased accumulation of tanshinones	[145]
*Salvia sclarea* L.	PKYLX71:35S vector containing DXS or DXR	Hairy roots	Acetone	Terpenoids	Enhanced biosynthesis of abietane diterpenes	[146]
*Brassica rapa* L.	pBI121S vector containing BraLTP2	Leaves	Methanol-water	Different metabolites	Upregulation of 43 different secondary metabolites.	[147]
*Lycium ruthenicum* Murr.	pCAMBIA1307-TCP4-OE	Hairy roots	Methanol	Different metabolites	higher relative abundances of different secondary metabolites	[148]
